# Comparative Study of the Perspectives on the Impact of a Culturally Responsive Picture Exchange Communication System for Children With Autism Spectrum Disorder in the United Arab Emirates

**DOI:** 10.3389/fped.2022.841064

**Published:** 2022-04-29

**Authors:** Mohammed Safi, Maxwell Peprah Opoku, Mariam Alshamsi, Ahmad Hassan Abu-shariha

**Affiliations:** ^1^Speech–Language Pathology, College of Medicine and Health Sciences, United Arab Emirates University, Al Ain, United Arab Emirates; ^2^Special Education Department, College of Education, United Arab Emirates University, Al Ain, United Arab Emirates

**Keywords:** parents, practitioners, autism spectrum disorder, communication, culture, United Arab Emirates, PECS, neurodevelopmental disorders

## Abstract

**Background:**

The Picture Exchange Communication System (PECS) is a widely accepted way to develop the communication skills of children with developmental disabilities such as autism spectrum disorder (ASD). However, the efficacy of PECS has been largely studied in Western societies and little research is available regarding its role in the development of children with ASD in a non-Western cultural context. The purpose of this study was to compare the parent and the practitioner perceptions of the use of PECS for children with ASD in the United Arab Emirates.

**Method:**

A new scale made up of three subscales was used to collect the data from the parents and the practitioners who were either related to or working with a cohort of children with ASD. The data were analyzed using Statistical Package for the Social Sciences (SPSS) and subjected to the computation of means, *t*-tests, analysis of variance (ANOVA), binary logistical regression, and two-way factorial ANOVA.

**Results:**

The results showed uncertainty or neutrality among the parents and the practitioners toward the use of PECS. Variables such as age, years of experience using PECS, and gender were noted to influence perceptions of PECS.

**Conclusion:**

The need for policymakers to consider developing regular training for parents and practitioners on the use of PECS and other implications are discussed.

## Introduction

Autism spectrum disorder (ASD) is the most common developmental disability among children (1, 2). Autism spectrum disorder is a neurodevelopmental disorder that affects individuals’ behavioral, communication, and social skills (2, 3). It is estimated that 30–121 in every 10,000 individuals are living with a form of ASD (2). In most instances, it is difficult to include individuals with ASD in school or societal activities because of the challenges associated with the disorder (4). For example, they may exhibit repetitive behavioral patterns that may disrupt their activities and interactions and may not usually be flexible regarding the changes in their activities (2, 3). Children with ASD have problems developing relationships, communicating, and engaging in social interactions with peers and other members of society (1, 2, 5–7). The compounding behavioral problems are the large number of children with ASD who struggle to develop language for effective communication with others (2). Indeed, one-third to nearly half of all children with ASD do not have normal language abilities (8), and 25–30% lack any form of language for functional communication (9–11). While the estimated number of those who lack adequate communication abilities among adults living with ASD is 50% (12), the lack of language and communication can have negative consequences on the efforts to promote the development and inclusion of children with ASD in education (13, 14). Thus, it is essential for the practitioners and the parents to have the means to help children with ASD develop their capacity for language and promote their communication skills. With this in mind, this study explored the perceptions of the practitioners and the parents of the use of picture exchange communication systems (PECS) to enhance the development of children with ASD in the United Arab Emirates (UAE).

Augmentative and alternative communication (AAC) has been identified as a useful means to assist language development in individuals with communication disorders, such as those living with ASD (12, 14–16). The AAC refers to the development of strategies to enable individuals with a communication disorder to overcome temporary or permanent impairment in communication (17, 18). The use of AAC not only helps individuals develop a form of language (5, 18) but also helps strengthen the relationships between individuals with disabilities and the other members of society (2, 10, 16). Although several AAC approaches have been used to develop the communication skills of children with ASD, PECS remains one of the most widely used AAC approaches for enhancing the ability of children with ASD who have severe language and communication delays to interact with others (1, 8, 18).

The PECS was developed to train children with ASD to interact with others (18, 19). The PECS training program, which emerged from an applied behavior analysis approach, is divided into six phases: (a) communication, (b) picture identification, (c) distance and persistence, (d) making sentences, (e) answering questions, and (f) commenting on various scenarios (18, 19). In using the PECS, the child with ASD will work with two adults who play the roles of physical prompter and communication partner. While the adult uses verbal communication to relay information, the child with ASD responds using a picture. The child with ASD is required to acquire competence in each phase of the training before moving to the next.

It has been consistently reported that the children with ASD who undergo each facet of the PECS training are able to communicate effectively with other members of society (20).

Indeed, a corpus of studies from countries such as Australia, Canada, the United Kingdom, and the United States shows that the use of PECS among children with ASD is closely linked to better communication and positive behavior (5, 6, 10, 21–23). Some studies have demonstrated that the use of PECS among children with ASD enables them to engage in prolonged conversation, initiate conversation, and make demands, decisions, and choices (5, 10, 24, 25). Furthermore, the use of PECS enables children with ASD to develop better or more positive behavioral skills that facilitate their inclusion in society (6, 24, 26). This clearly underscores the need for the practitioners and the parents to adopt PECS to enable non-verbal children with ASD to develop a form of language for communication.

Unfortunately, the studies on the impact of PECS have mainly been limited to the “advanced” societies, with the contribution of PECS in the lives of children with ASD in non-Western societies largely going unresearched. For example, Hume et al. (1) suggested the need for future studies to consider assessing the influence of ethnicity on interventions for children with ASD. In countries in the Middle East and North Africa (MENA) region, the Arab culture and the Islamic religious background play major roles in shaping the daily living experiences of the children and their families. The local cultural attitudes and perspectives influence communication abilities in neurotypical children, as well as those who have communication impairments (13, 14). Hence, for the PECS to promote children’s development, the training and PECS program ought to adhere to the local context and cultural perspectives. In this study, an attempt was made to understand whether PECS training developed based on the local cultural practices could have a positive impact on the development of children with ASD in the UAE.

### Family Centered Rehabilitation Care and Practices

The contemporary approaches to rehabilitation and healthcare are moving toward family centered care and practices (27). Historically, the practitioners were the main drivers of rehabilitation and service provision to individuals with disabilities and their families (28). Here, the professional would decide on the care needed and rarely involve the family in the provision of services. However, in an effort to promote an inclusive society, the family has been identified as a formidable institution that can play a central role in rehabilitation care (28, 29). Strong arguments have recently been advanced in favor of giving the family a role in the rehabilitation of their members (27). It has been suggested that the collaboration between the practitioners and the families is a good step toward promoting effective care services for children with disabilities (27, 28). In particular, the families live with their members who have disabilities and can pass vital information to the practitioners that can be used in the rehabilitation process (14, 16, 27). While the practitioners may focus their attention on the etiology of the challenges faced by the child and on effective intervention methods, the family can continue the rehabilitation process by implementing best practices at home (14, 17, 28). This clearly underscores calls for close collaboration between the practitioners and the families (14, 17, 29) in an attempt to develop the skills of children with developmental disabilities such as ASD.

Previously, attempts were made to examine the perceptions of the parents and the practitioners of the implementation or effectiveness of PECS for children with ASD (22–25, 30–35). Most of these studies reported that the practitioners and parents were favorably disposed toward the use of PECS to develop the communication skills of children with ASD. Most importantly, they reported its usefulness and ease of use in promoting developmental skills among children with ASD. A United States-based study explored the perceptions of the practitioners of the use of PECS among children with ASD (32). While reporting a positive impact on children, they reported differences between various types of practitioners working with children with ASD. For example, they noted that the teachers were more positive about the importance of PECS despite their perception of greater barriers to the use of PECS compared to therapists. Therapists, however, were more positive about the effectiveness of PECS in developing communication. Another study reported parents’ perception of the impact of PECS on developmental skills among children with ASD (31). They noted the following differences between the parents with respect to their knowledge and income levels: the higher the education and income of the parents, the more knowledgeable they were regarding the use of PECS. These studies (31, 32), however, focused on the impact of PECS on the development of children generally without focusing on specific developmental domains such as learning skills or social interaction. Children with ASD struggle in several domains, such as behavior, language, and social skills (2, 3). Therefore, it is vital for future comparative studies, such as the one reported here, to focus on the perceptions of the practitioners and the parents across these domains.

### Contextualization

The United Arab Emirates (UAE) is a federal state consisting of seven emirates (Abu Dhabi, Ajman, Fujairah, Ras Al Khaimah, Sharjah, and Umm Al Quwain). The UAE is a multicultural society located in the Gulf region of Western Asia (36). It has an estimated population of 9.6 million, and oil and tourism are the main drivers of the national economy (37). The disability is traditionally understood as an act of God, and thus there is a consensus that society needs to support those living with any form of impairment (38, 39). As a part of an effort to achieve an inclusive society, the federal government of the UAE has developed several policies geared toward maximizing the potential of individuals with disabilities, such as those with ASD (38–42). Although there are no official recent data on the population of individuals with ASD in the UAE is available, it is believed that a sizable number of children in the UAE have been diagnosed with ASD.

In 2006, the passage of the Convention on the Rights of Persons with Disabilities fueled domestic legislation in the UAE. For example, Federal Law Number 29 of 2006, Federal Decree Number 116 of 2009, and Law Number 2 of 2014 are key milestones in the development of the legal structure relevant to children with ASD in the UAE (36). These legal instruments exhort the parents and the practitioners to provide effective care services to individuals with disabilities such as ASD. In terms of health, the practitioners from both public and private healthcare facilities are encouraged to adopt best practices to enhance the well-being of the persons such as those with ASD. In terms of education, the facilities such as rehabilitation centers have been established to ensure that all children with ASD receive personal skills training to allow them to contribute toward the overall development of the country (40–42). Although some gains have been made in terms of collaboration between the parents and the practitioners, the body of research on any given intervention and its impact on the development of children with ASD is very small. With PECS being widely identified as useful for promoting the development of children with ASD (18–22), it would be useful to develop insights into its effectiveness in a MENA cultural environment such as that of the UAE.

The purpose of this study was to compare the perceptions of the parents and the practitioners involved in the provision of service to children with ASD on the impact of PECS. The study is vital at a time when the government of the UAE is exploring ways to enhance the development of all children, including those with ASD. This study will provide baseline information about the usage of PECS and its impact among children with ASD in the UAE, as well as potential areas to strengthen during development and future training. To achieve the objectives of this study, it was guided by the following questions:

– What is the association between demographic variables and perceptions of PECS?
– What factors predict the likelihood that the parents or the practitioners would report that they had a positive perception of the impact of PECS?
– Do the parents or the practitioners moderate the relationship between other demographic variables and perceptions of PECS?

## Materials and Methods

### Study Participants

The participants in this study were the parents and the practitioners working with a cohort of students with ASD across three of the UAE’s seven emirates. The participants were recruited from the emirates of Abu Dhabi, Dubai, and Sharjah. Multiple types of facilities involved in the education of children with ASD—centers for persons of disabilities (determination), general schools, and early childhood centers—in the three emirates were chosen based on the convenience and availability of participants to take part in the study. The practitioners included teachers, special education teachers, and therapists. The participants were included in the study if they satisfied the following inclusion criteria: (a) they had received training in the use of PECS; (b) they were working with or caring for children with ASD between 0 and 18 years of age or had done so previously; and (c) they consented to participate in the study. Likewise, the following criteria guided the recruitment of parents: (a) they had a child with ASD enrolled in the selected facility; (b) their child was between 0 and 18 years old; (c) they had received training in the use of PECS; and (d) they consented to take part in this study. Consequently, the participants were excluded if they were not parenting a child with ASD or were not a practitioner at the selected rehabilitation center. Moreover, the participants were excluded if they had not received training in the use of PECS.

A total of 144 participants took part in this study. The demographic characteristics of the participants in this study are summarized in Tables 1, 2. A total of 53% of the participants were parents and 47% were the practitioners (such as teachers, special education teachers, and therapists). A total of 51% of the participants were female and 49% were male. Regarding the children with ASD, 60% were boys and 40% were girls; 51% were between the age group of 1–5 years; and 49% were at least 6-years old. Moreover, 40% of the children were enrolled in inclusive schools, compared to 34% and 26% who were enrolled in early childhood centers and special schools, respectively (see Tables 1, 2 for more details).

**TABLE 1:** Summary of the association between two-level demographics and perceptions.

Category (*N* = 144)	Sample	Comm. Skills	Learning skills	Social inter. Skills	Overall PTPECS
***Participants (n = 139)***					
Parents	73 (53%)	5.09 (0.57)	5.12 (1.01)	5.18 (0.45)	5.13 0(0.46)
Practitioners	71 (47%)	3.39 (1.01)	3.49 (0.53)	3.74 (1.04)	3.55 (0.89)
*t*		11.97#**	12.40#**	10.40#**	12.88#**
partial eta squared		0.50	0.54	0.47	0.56
***Gender (n = 139)***					
Male	68 (49%)	4.99 (0.74)	4.99 (0.74)	5.11 (0.59)	5.04 (0.64)
Female	71 (51%)	3.60 (1.11)	3.72 (1.05)	3.91 (1.110)	3.76 (1.00)
*t*		8.69#**	8.22#**	8.10#**	9.04#**
partial eta squared		0.33	0.32	0.26	0.37
***Gender of children with ASD (n = 139)***					
Boy	83 (60%)	4.32 (1.18)	4.36 (1.12)	4.54 (1.03)	4.42 (1.04)
Girl	56 (40%)	4.22 (1.17)	4.31 (1.10)	4.45 (1.13)	4.34 (1.09)
*t*		0.50	0.25	0.49	0.61
partial eta squared		0.003	0.001	0.006	0.001
***Years supporting child with ASD (n = 124)***					
1–5 years	63 (51%)	4.08 (1.32)	4.14 (1.28)	4.34 (1.21)	4.19 (1.21)
6 years or more	61 (49%)	4.57 0 (0.92)	4.66 (0.78)	4.78 (0.74)	4.68 (0.74)
*t*		–2.40#**	–2.76#** 0.07	–2.49#** 0.07	–2.70#** 0.07
partial eta squared		0.06			
***Years supporting children using PECS (n = 139)***					
1–5 years	79 (57%)	4.33 (1.19)	4.41 (1.14)	4.56 (1.05)	4.44 (1.05)
6 years or more	60 (43%)	4.23 (1.16)	4.26 (1.07)	4.42 (1.10)	4.31 (1.06)
*t*		0.48	0.81	0.81	0.79
partial eta squared		0.001	0.005	0.002	0.004
***Frequency of using PECS (n = 139)***					
Rarely	51 (37%)	4.15 (1.23)	4.20 (1.12)	5.28 (1.11)	4.26 (1.08)
Frequently	88 (63%)	4.36 (1.14)	4.43 (1.10)	4.57 (1.04)	4.46 (1.04)
*t*		–0.99	–1.14	–0.96	–1.08
Partial eta squared		0.007	0.009	0.009	0.008

**p < 0.01; PECS, Picture Exchange Communication System; #, violation of assumption of homogeneity of variance with results of within groups reported; PTPECS, Perception toward Picture Exchange Communication Scale; Not all participants provided their demographic information.

**TABLE 2:** Summary of the association between three-level demographics and perceptions.

Category (*N* = 144)	Sample (%)	Comm. Skills	Learning skills	Social inter. Skills	Overall PTPECS
***Institution (n = 139)***					
Inclusive school	55 (40%)	4.18 (1.18)	4.22 (1.11)	4.49 (1.13)	4.24 (1.10)
Special school	36 (26%)	4.22 (1.19)	4.21 (1.20)	4.30 (1.13)	4.26 (1.12)
Early childhood center	48 (34%)	4.43 (1.12)	4.59 (1.00)	4.75 (1.11)	4.63 (0.93)
*F*		0.64	1.77	2.81*	2.07
Partial eta squared		0.009	0.03	0.04	0.03
***Age of children with ASD (n = 143)***					
1–5 years		4.07 (1.21)	4.07 (1.18)	4.36 (1.10)	4.21 (1.06)
6–10 years	21 (15%)	4.31 (1.21)	4.46 (1.14)	4.49 (1.10)	4.43 (1.10)
11 or more years	50 (35%)	4.32 (1.12)	4.35 (1.06)	4.49 (1.06)	4.40 (1.03)
*F*	72 (50%)	0.39	0.87	0.13	0.34
Partial eta squared		0.006	0.01	0.002	0.005
***Age child began using PECS (n = 139)***					
0–2 years	49 (35%)	3.83 (1.25)	3.99 (1.25)	4.10 (1.25)	3.99 (1.18)
3–5 years	67 (48%)	4.32 (1.12)	4.33 (1.04)	4.49 (0.98)	4.41 (0.99)
6 or more years	23 (17%)	5.12 (0.22)	5.15 (0.22)	5.20 (0.32)	5.16 (0.19)
*F*		37.59#**	32.44#**	26.98#**	37.84#**
Partial eta squared		0.14	0.12	0.12	0.14

*p < 0.05; **p < 0.01; ASD, Autism Spectrum Disorder; PECS, Picture Exchange Communication System; #, violation of the assumption of homogeneity of variance with Welch statistic reported; PTPECS, Perception toward Picture Exchange Communication Scale; Not all participants provided their demographic information.

### Instrument

A two-part questionnaire was used for data collection. The first part of the instrument collected information about the demographic characteristics of the participants who took part in this study. These cover background information such as the category of the participant (the parent or the practitioner), gender, gender of the child with ASD, age of the child with ASD, years of experience supporting children with ASD, years of experience supporting children with ASD using PECS, frequency of using PECS, the institution of enrollment, and age at which the child began using PECS.

The second part of the instrument was the Perception toward Picture Exchange Communication Scale (PTPECS), which was designed for this study. This instrument was designed based on a literature review of areas of support required by children with ASD in education (communication, learning, and social interaction) (5, 17, 18, 20, 22, 25, 32). The PTPECS is made up of 16 items reflecting 3 factors (communication skills, learning skills, and social interaction skills), which are anchored on a 6-point response scale, ranging from 0 (not applicable) to 6 (strongly agree). The researchers reported the mean scores for the participants, which were computed as the sum mean divided by the number of items. A mean score of at least 5 was interpreted as a favorable perception toward PECS.

The first subscale, on communication skills, is made up of five items with some examples as follows: “In my opinion, PECS helps the child with autism express his desires and needs,” “Based on my experience, PECS helps the child with autism express his feelings,” and “In my view, PECS stimulates the child with autism to improve their spoken language skills.” The second subscale, learning skills, is made up of five items, with some examples as follows: “Based on my experience, PECS helps the child with autism improve their performance in academic subjects (mathematics, reading),” “In my opinion, PECS helps the child with autism learn appropriate behaviors,” and “In my opinion, PECS encourages the child with autism to learn new vocabulary.” The third subscale, social interaction skills, is made up of six items, with some examples as follows: “In my opinion, PECS helps the child with autism initiate interactions with peers (make new friends and play with them),” “Based on my experience, PECS helps the child with autism improve their group participation skills,” and “In my opinion, PECS is the best way to improve social skills in children with autism.”

The scale was subjected to content validation following the Delphi approach, i.e., sharing the draft scale with experts to review it (43). A draft scale containing items in English and Arabic was given to three academics, three practitioners, and three parents who are fluent in both languages to determine whether the items on the scale were appropriate, accurate, clear, and unambiguous. The initial draft contained 25 items. However, following the reviewers’ recommendations, the scale was revised to include 19 items. The factor structure of the scale was determined using factor analysis. However, three items were deleted as they had poor factor loadings, thus leaving 16 items for analysis. Afterward, the reliability of the scale was assessed using Cronbach’s alpha and yielded the following scores: Overall PTPECS (0.96), communication skills (0.88), learning skills (0.87), and social interaction skills (0.92).

### Procedure

The study and its protocols received ethical approval from the institutional review board at United Arab Emirates University (ERS_2021_7331). Afterward, the formal emails were sent to schools (regular schools, centers for rehabilitation of persons with autism, and early childhood centers) providing services to children with autism across the three emirates (Abu Dhabi, Dubai, and Sharjah). Additionally, the practitioners working with children with ASD and/or their parents were contacted directly.

Due to the outbreak of coronavirus disease 2019 (COVID-19), the data were collected online using Google Forms from 15 January 2021 to 1 April 2021. The Google Forms questionnaire contained both Arabic and English versions of the statements. The participants were assured that neither their identity nor that of their children or the institution within which their children were enrolled would be shared with anyone outside the research team. They were informed that there was no reward or reimbursement associated with participation in the study. All participants provided informed consent prior to accessing the scale.

### Data Analysis

The data were transferred from Google Forms to Microsoft Excel before cleaning by the first author. After the cleaning, the data were transferred to SPSS version 26 for analysis. The missing data for the PTPECS were less than 5%; thus, we used the expectation-maximization algorithm to impute the missing data. The data were then checked to ensure that they would not violate the assumption of normality. The results from the normality test, especially the histogram, showed that the data were normally distributed for parametric tests (44).

We checked the mean scores for the scale before continuing to answer the research questions. To answer Research Question 1, we computed *t*-tests and analysis of variance (ANOVA) to ascertain the association between demographic profiles and perceptions toward PECS. While *t*-tests were computed for the demographic variables with two levels, ANOVA was computed for the demographic variables with three or more levels. Here, we checked the homogeneity of variance using Levene’s test to ensure that they were not violated. In the event of a violation of Levene’s test with respect to the calculation of *t*-tests, the results of the within-groups as recommended by Pallant (43) were reported. On the ANOVAs, in the event of a violation of the homogeneity of variance tests, the results of the Welch statistic, as suggested by Pallant (44), were reported. For the *t*-tests and ANOVA, the magnitude of the weight of the difference (effect size) between the participants was computed using partial eta squared, which was interpreted as follows: small (0.01–0.05), moderate (0.06–0.09), and large (at least 0.1) (44).

To answer Research Question 2, bivariate logistic regression was used to ascertain the likelihood that the parents or the practitioners would have a positive perception of PECS. The results showed that the model satisfied the goodness-of-fit test (omnibus tests of model coefficient = 138.08, *df* = 11, *p* = 0.001). Also, the Hosmer–Lemeshow goodness-of-fit test also showed that the data fit the model well (*Chi-square* = 10.76, *df* = 8, *p* = 0.22). The model for the logistic regression was appropriate, as explained by the two goodness-of-fit tests.

To answer Research Question 3, we used a two-way ANOVA to understand the interaction between the category of participants (the practitioner or the parents), other demographic features, and perceptions of PECS. Once again, we checked the homogeneity of variance tests to ensure that they were not violated.

## Results

The overall mean score for the participants was 4.38 (*SD* = 1.06). The mean scores for the three subscales were as follows: communication skills (*M* = 4.28; *SD* = 1.16), learning skills (*M* = 4.35; *SD* = 1.11), and social interaction skills (*M* = 4.47; *SD* = 1.07).

### Association Between Demographics and Perception Toward Picture Exchange Communication Systems

Independent-sample *t*-tests were conducted to compare the association between two-level background variables and perceptions of PECS (see Table 1). There were significant differences among participants’ views of the usefulness of PECS along the lines of the type of participant, gender, and the number of years of experience supporting children with ASD. First, the results showed a significant association between the type of participants and overall perceptions of PECS, *t* (137) = 12.29, *p* = 0.001, with a small moderate effect size (partial eta squared = 0.56). Specifically, the mean scores showed that parents were more positive about the impact of PECS on their children with ASD than the practitioners. Similar trends were observed on all three subscales.

Second, on gender, there was a significant difference among the participants in terms of their perceptions of PECS, *t* (137) = 9.04, *p* = 0.001, with a small effect size (partial eta squared = 0.37) (see Table 2). The mean scores showed that males were more positive about the impact of PECS on children with ASD than females. Once again, similar trends were observed on all three subscales.

Third, there was a significant association between the number of years supporting children with ASD and perceptions of PECS, *t* (122) = -2.70, *p* = 0.008, with a small effect size (partial eta squared = 0.07). The mean scores showed that participants who indicated they had supported children with ASD for more years tended to have more positive perceptions of PECS than those who indicated otherwise. Again, similar trends were observed on all three subscales.

A one-way between-groups ANOVA was conducted to explore the association between three-level demographics and perceptions of PECS. There was a statistically significant difference in terms of the age at which the participants indicated the children had begun using PECS and their perceptions of PECS, *F* (2, 136) = 37.59, *p* = 0.001, with a small effect size (partial eta squared = 0.14). The *post hoc* comparison using Tukey’s HSD test showed that participants who indicated that their children or the children they were working with began using PECS when they were older than six differed from others who indicated otherwise. Similar trends were observed between participants on the other three subscales.

There was also a statistically significant difference between participants by institution but only on one subscale, social interaction skills, *F* (2, 140) = 2.81, *p* = 0.05, with a small effect size (partial eta squared = 0.04). The *post hoc* comparison using Tukey’s HSD comparison test found no significant difference between participants.

### Predictors of Perceptions of Picture Exchange Communication Systems

A direct logistic regression was performed to assess the likelihood of the parents or the practitioners reporting positive attitudes toward PECS (see Table 3). The full model was statistically significant, χ^2^ (11, *N* = 144) = 138.08, *p* = 0.001. The model as a whole explained 66% (Cox and Snell R square) and 89% (Nagelkerke R square) of the variance in the status of parents’ or the practitioners’, respectively, as well as correctly identifying 96.1% of the cases. Individually, only three independent variables made significant contributions in the category of participants (gender, number of years supporting a child using PECS, and learning skills). The strongest predictor of perception of PECS was gender, recording an odds ratio of 219.45. This indicated that there is an over 200% chance that the parents’ or the practitioners’ perceptions of PECS could be influenced by their gender. Also, an odds ratio of 14.95 was recorded for the number of years of experience supporting a child using PECS, indicating that for every increase in the number of years of experience supporting a child using PECS, there was a just over 14% chance that the participants would report that they felt that PECS has a positive impact on the development of children with ASD. Moreover, the odds ratio for learning skills was 0.59, which is less than 1, which means that for every increase in learning skills, participants were 0.59 times less likely to report a positive impact of PECS on children with ASD.

**TABLE 3:** Summary of binary logistic regression.

Variable	B	SE	OR	95% CI Lower Upper	Wald Statistic	*p*
Gender	5.39	1.70	219.45	[0.001 – 0.114]	10.03	0.002**
Years supporting children with autism	0.34	1.06	1.45	[0.18–11.15]	0.10	0.75
Years supporting a child using PECS	2.81	1.55	14.95	[0.80–343.16]	3.42	0.05*
Institution	0.100	0.64	1.10	[0.32–3.86]	0.02	0.88
Gender of child	–0.97	1.06	0.38	[0.05–3.01]	1.06	0.36
Age of child	–1.85	0.100	0.16	[0.02–1.11]	3.45	0.06
Age child started using PECS	–1.57	0.98	0.21	[0.03–1.44]	2.53	0.21
Frequency of using PECS	0.81	1.35	2.24	[2.24–0.16]	0.36	0.55
Communication skills	0.05	0.14	1.05	[1.05–0.79]	0.12	0.73
Learning skills	–0.53	0.20	0.59	[0.400–0.87]	7.08	0.008**
Social interaction skills	–0.100	0.19	0.90	[0.62–1.33]	0.26	0.61

*p < 0.05; **p < 0.01; PECS, Picture exchange communication system.

### Interaction Between the Type of Participant and Perceptions of Picture Exchange Communication Systems

A two-way ANOVA was conducted to understand the impact of participant type and other demographics on perceptions of PECS (see Table 4). Regarding overall perceptions of PECS, there was an interaction effect between participant type and the number of years of experience supporting children with ASD, *F* (1, 124) = 11.75, *p* = 0.001, with a moderate effect size (partial eta squared = 0.09). The mean scores showed that participants who indicated they had more years of experience supporting children with ASD were inclined to have a more positive perception of the usefulness of PECS than those who indicated otherwise.

**TABLE 4:** Two-way analyses of variance for participant type, other demographics, and perception.

Variable	*MS*	*F*	*p*	η^2^
***Communication skills***				
Gender	5.70	0.34	0.56	0.002
Years supporting children with ASD	138.23	9.57	0.003**	0.07
Years supporting children with PECS	21.15	1.27	0.26	0.009
Institution	3.48	0.21	0.81	0.003
Gender of child	4.63	0.28	0.60	0.002
Age of child	5.03	0.30	0.74	0.004
Age child started using PECS	63.42	3.90	0.05*	0.03
Frequency using PECS	4.01	0.24	0.63	0.002
***Learning skills***				
Gender	0.24	0.02	0.90	0.001
Years supporting children with ASD	149.08	11.74	0.001**	0.09
Years supporting children with PECS	10.19	0.71	0.40	0.005
Institution	7.78	0.54	0.58	0.008
Gender of child	0.80	0.06	0.82	0.001
Age of child	0.86	0.06	0.94	0.001
Age child started using PECS	32.27	2.27	0.13	0.02
Frequency using PECS	0.98	0.07	0.79	0.001
***Social interaction skills***				
Gender	0.25	0.01	0.92	0.001
Years supporting children with ASD	135.06	6.93	0.01**	0.05
Years supporting children with PECS	1.11	0.05	0.83	0.001
Institution	8.09	0.37	0.69	0.005
Gender of child	20.00	0.90	0.34	0.006
Age of child	18.50	0.82	0.44	0.01
Age child started using PECS	100.49	4.59	0.03*	0.03
Frequency using PECS	30.56	1.38	0.24	0.01
***Total PTPECS***				
Gender	46.30	0.37	0.55	0.003
Years supporting children with ASD	1264.53	11.75	0.001**	0.09
Years supporting children with PECS	29.69	0.23	0.63	0.002
Institution	17.39	0.14	0.71	0.001
Gender of child	45.95	0.36	0.70	0.005
Age of child	45.95	0.36	0.70	0.005
Age child started using PECS	569.17	4.60	0.03*	0.03
Frequency using PECS	112.65	0.90	0.34	0.007

*p < 0.05; **p < 0.01; ASD, Autism Spectrum Disorder; PECS, Picture Exchange Communication System; PTPECS, Perception toward Picture Exchange Communication Scale.

Furthermore, there was an interaction effect between participant type and the age when the child started using PECS, *F* (1, 134) = 4.60, *p* = 0.03, with a small effect size (partial eta squared = 0.03). The *post hoc* comparison using Tukey’s HSD comparison test showed that participants who indicated that the children with ASD started using PECS when they were at least 6 years were different from those who indicated otherwise.

On the subscales, with the exception of learning skills, there were interaction effects between participant type, years supporting children with ASD, and the age when the children began using PECS. For example, with respect to communication skills, there was an interaction effect between the participant type and the number of years of experience supporting children with ASD, *F* (1, 127) = 9.19, *p* = 0.003, with a moderate effect size (partial eta squared = 0.07). The participants with more years of experience supporting children with ASD indicated that they were more positive regarding the impact of PECS on communication skills than those who indicated that they had fewer years of experience supporting children with ASD.

There was also an interaction effect between the participant type and the age at which the child had started using PECS, *F* (1, 138) = 3.90, p = 0.05, *p* = 0.03, with a small effect size (partial eta squared = 0.03). The *post hoc* comparison using the Tukey HSD test revealed that participants who indicated that their children or the children they were working with had begun using PECS when they were at least 6 years old tended to have a more positive view of its impact on communication skills than the others.

## Discussion

This study attempted to compare the perceptions of the parents and the practitioners on the impact of PECS on the development of children with ASD in the UAE. The study focused on three important domains: communication skills, learning skills, and social interaction skills. However, the findings of the study show that all participants were ambivalent about the impact of PECS on the development of children with ASD. This finding is somewhat inconsistent with previous studies that reported favorable attitudes on the part of parents and practitioners toward the impact of PECS on the development of children with ASD (21, 31, 32). The findings reported here may be attributed to multiple factors: (a) a lack of consensus on how to identify children with ASD; and (b) a lack of agreement in the UAE on what constitutes an effective curriculum for educating children with ASD. Like any other non-Western society, little has been done in terms of determining ways to identify individuals with ASD in the UAE. In view of this, the practitioners and the parents in the UAE may lack sufficient knowledge of the various characteristics of ASD, as well as of how to determine whether individuals with ASD are making positive progress in their communication and language development. It is not surprising that there are limited studies on the identification and management of ASD within the cultural context of the UAE. The results of this study indicate a need for stakeholders in the UAE to develop a contextual understanding of the characteristics of ASD and of suitable interventions. In this way, stakeholders can determine whether the interventions being implemented are having an impact on the children.

Furthermore, the findings show differences between the parents and the practitioners in their perceptions of the usefulness of PECS with respect to all three domains: communication skills, learning skills, and social interaction skills. This finding is inconsistent with a previous intervention study that reported an unfavorable disposition toward continual use of PECS for the development of children with ASD among participating families (17). In the UAE context, two reasons could explain this finding. First, it has been argued that children spend more time with their parents than with their practitioners (14). It may be that the parents had continued to use PECS at home and may have observed their children’s ongoing development. In view of this, they may have witnessed their children being able to use PECS to communicate and learn, as well as to interact socially with others. Second, it is possible that the training given to the practitioners on the use of PECS may have been on a once-off or irregular basis. This could have affected their ability to continue using PECS to help the development of students with ASD. It has been suggested that parents may spend time researching and discovering the best ways to support their children (13). It is apparent that after completing training, parents may have continued to explore other learning avenues that have enabled them to become proficient users of PECS. This may not be the case for the practitioners who have more students to support on a day-to-day basis. This finding may indicate a need for further investigation of possible innovative ways to provide the practitioners and the parents with training programs in an effort to promote the development of children with ASD in the UAE.

One demographic variable that emerged as an important predictor of perceptions of PECS was gender. Males demonstrated favorable perceptions toward the usage or importance of PECS in terms of the development of children with ASD compared to females in this study. This finding appears to be the first time that the influence of gender has emerged as a predictor of perceptions of the impact of PECS on the development of children. This is unsurprising because, in the culture of the UAE, gender plays an important role in people’s development in that there are gender norms that determine the role of males and females in society. However, even with education and training in the usage of PECS, males indicated a more favorable disposition toward PECS than females. In an effort to design training programs for the stakeholders (such as the parents and teachers who took part in this study) involved in the development of children with ASD in the UAE, educators should consider trying to build the efficacy of all participants. For example, they should consider building the confidence of both male and female practitioners and parents who are directly involved in the development of children with ASD. This has the potential to enhance the development of children with ASD because parents and practitioners can play a substantial role in their development regardless of their gender.

The participants’ total years of experience supporting children with ASD also emerged as an important variable that provides additional insights into perceptions of PECS. The findings of the study showed that the more years of experience both parents and practitioners had supporting children with ASD, the more likely they were to have a positive perception of the importance of PECS compared to those who had fewer years’ experience supporting children with ASD. Similarly, the findings of the study showed that the more the years the participants indicated they had supported a child using PECS, the more likely they were to have a positive perception of the usefulness of PECS to children with ASD. Once again, this appears to be the first time that the number of years the participants had supported children with ASD and the number of years they had used PECS have been focused on in research on PECS. This finding may be of particular interest to policymakers or educators in the UAE who are exploring effective ways to make quality education accessible to children with ASD. In particular, the knowledge of more experienced parents or practitioners could be tapped to enhance positive services for children with ASD. Collaboration between stakeholders has been recognized as vital in special education (14). Experienced practitioners or parents may be empowered to run regular professional development training activities for less experienced ones. This could be an important learning experience for novice parents or practitioners who could benefit from the experienced ones’ knowledge.

Another variable that provides additional understanding of the perceptions of parents and practitioners of PECS was the age at which the children with ASD began using PECS. Specifically, the findings showed that participants who indicated that the children with ASD began using PECS when they were at least 6 years old were more positive about its impact on their development than those who indicated otherwise. This finding is surprising because it has been proposed that early intervention is fundamental in helping children with special needs, such as those living with ASD (13, 14). It has usually been recommended that for children with ASD to benefit from any intervention, such as the use of PECS, there is a need for them to be exposed to the intervention at an early age (14). It appears that the finding of this study did not support such a proposition, as children who began using PECS at a relatively older age seemed to be showing positive progress in the domains of communication and social interaction skills. Perhaps, in the situation of the children with ASD in the UAE, the older they were when they began to use interventions such as PECS, the more appreciable development they would show. It is apparent that older children with ASD may be more mature and may understand instructions more effectively than younger children with ASD. However, more intervention studies may be needed before a conclusion could be made regarding the age at which children began using PECS and its impact on their development.

### Study Limitations

The findings of this study should be interpreted with caution because of several limitations. First, the participants were made available by the institutions. There is the potential for bias, as the institutions may not have provided a list of all potential participants to the research team. However, the research team provided a detailed explanation to the parents and the practitioners before they took part in this study. It is likely that they provided accurate responses to the items on the scale corresponding to the development of their children. Second, it was beyond the scope of the study to determine whether the children had been diagnosed with ASD or were living with another comorbidity condition(s). Indeed, since the schools and centers worked directly with the parents and other allied institutions, they are in a better position to identify children who have been diagnosed with ASD. Third, the data used in the reporting of this study were collected from three of the UAE’s seven emirates, so they may not be representative of the views of all the parents and the practitioners working with children with ASD. However, there is a strong sense of a shared culture in the UAE, and as such, it is possible that the parents and the practitioners in the UAE may share a particular understanding or perception of ASD, meaning that the findings may be a mirror what patterns in other Emirates or similar contexts. Fourth, the study relied on the assessment by the parents and the practitioners and was thus unable to verify whether the various competencies had in fact been achieved by the children with ASD. Since the parents and the practitioners were trained to use PECS, they may have provided appropriate responses to the items on the scale. Moreover, the items were provided to the participants in both Arabic and English to ensure that they understood the statements and responded appropriately.

## Conclusion and Policy Implications

Both the family and the practitioners play vital roles in efforts toward the rehabilitation of children with disabilities such as ASD (27–29). The main aim of this study was to compare the perceptions of the parents and the practitioners toward the impact of PECS on the development of children with ASD in the UAE. The studies on the importance of PECS have mainly been conducted in Western societies. This study has made a substantial contribution to the literature by adding the perceptions of parents and practitioners toward the usage of PECS with children with ASD in the UAE. An appropriate tool, encompassing the domains of communication, learning skills, and social interactions skills, was developed and used to collect data from both parents and practitioners. The results showed the ambivalence of parents toward the impact of PECS on the development of children with ASD in the UAE. Also, a significant difference was found between the parents and the practitioners, with the former having a more positive view of the impact of PECS than the latter. Also, variables such as gender, years of experience supporting a child with ASD, years of experience supporting a child using PECS, and age at which the child with ASD began using PECS provided useful insights into the perception of the impact of PECS on the development of children with ASD. This study has made a substantial contribution to the literature in terms of documentation of the perceptions of parents and practitioners of the impact of PECS on the development of children with ASD in the UAE.

The results may provide a useful guide for policymakers involved in the promotion of education and development of children with ASD in the UAE. Indeed, the UAE government has made clear its intention to enhance the development of individuals with disabilities, including those with ASD, in the country. In the efforts toward policy reformation, consideration should be given to developing clear guidelines regarding ASD, its contextual understanding and characteristics, and educational pathways for children with ASD. This could serve as a reference point and basis for measuring the development of children with ASD in the UAE. Second, there is a need for more professional development for both parents and practitioners in the usage of PECS. The training could take into consideration the importance of building the capacity of all, regardless of gender, to contribute to the development of children with ASD. Also, policymakers may consider using more experienced parents and practitioners in the provision of professional development for upcoming or younger ones. They could pass vital experience they have acquired through education, as well as by working with children with ASD, to the novice parents and practitioners. The use of experienced practitioners and parents could also be a reliable source of regular training for inexperienced parents and practitioners. These recommendations, if considered, could help both practitioners and parents acquire relevant information to optimize the benefits associated with the use of PECS by children with ASD.

## Data Availability Statement

The raw data supporting the conclusions of this article will be made available by the authors, without undue reservation.

## Ethics Statement

The studies involving human participants were reviewed and approved by Research Office, United Arab Emirates University. The patients/participants provided their written informed consent to participate in this study.

## Author Contributions

MS, MO, MA, and AHA: conceptualization, investigation, resources, writing – original draft, and writing and reviewing the draft. MA: data curation and project administration. MO: formal analysis, methodology, and software. All authors contributed to the article and approved the submitted version.

## Conflict of Interest

The authors declare that the research was conducted in the absence of any commercial or financial relationships that could be construed as a potential conflict of interest.

## Publisher’s Note

All claims expressed in this article are solely those of the authors and do not necessarily represent those of their affiliated organizations, or those of the publisher, the editors and the reviewers. Any product that may be evaluated in this article, or claim that may be made by its manufacturer, is not guaranteed or endorsed by the publisher.
